# Perspective on design and technical challenges of Li-garnet solid-state batteries

**DOI:** 10.1080/14686996.2021.2018919

**Published:** 2022-01-18

**Authors:** Kostiantyn V. Kravchyk, Maksym V. Kovalenko

**Affiliations:** a Laboratory for Thin Films and Photovoltaics, Department of Advanced Materials and Surfaces, Empa – Swiss Federal Laboratories for Materials Science and Technology, Dübendorf, Switzerland; b Laboratory of Inorganic Chemistry, Department of Chemistry and Applied Biosciences, ETH Zürich, Zürich, Switzerland

**Keywords:** Li-garnet solid-state batteries, LLZO solid-state electrolyte, Li metal anode, energy density, charge-storage capacity, 50 Energy Materials, 206 Energy conversion, transport, storage, recovery < 200 Applications, 207 Fuel cells, Batteries, Super capacitors < 200 Applications

## Abstract

Solid-state Li-ion batteries based on Li-garnet Li_7_La_3_Zr_2_O_12_ (LLZO) electrolyte have seen rapid advances in recent years. These solid-state systems are poised to address the urgent need for safe, non-flammable, and temperature-tolerant energy storage batteries that concomitantly possess improved energy densities and the cycle life as compared to conventional liquid-electrolyte-based counterparts. In this vision article, we review present research pursuits and discuss the limitations in the employment of LLZO solid-state electrolyte (SSE) for solid-state Li-ion batteries. Particular emphasis is given to the discussion of pros and cons of current methodologies in the fabrication of solid-state cathodes, LLZO SSE, and Li metal anode layers. Furthermore, we discuss the contributions of the LLZO thickness, cathode areal capacity, and LLZO content in the solid-state cathode on the energy density of Li-garnet solid-state batteries, summarizing their required values for matching the energy densities of conventional Li-ion systems. Finally, we highlight challenges that must be addressed in the move towards eventual commercialization of Li-garnet solid-state batteries.

## Graphical abstract

**Figure:**
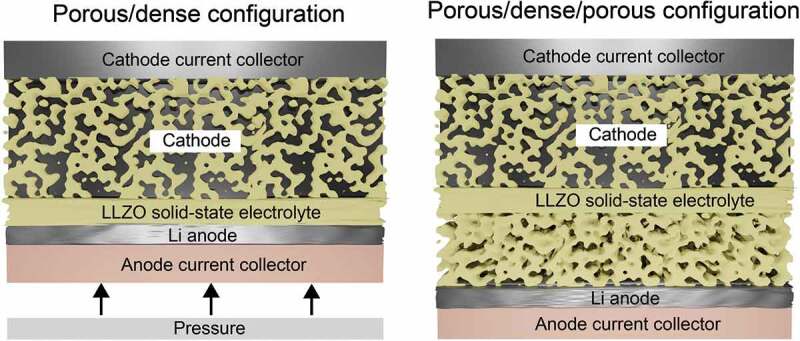

## Introduction

Li-ion batteries based solely on solid-state electrolytes (SSEs) are of great interest given the anticipated advantages in safety, working temperature range, improved gravimetric, and volumetric energy density as compared to conventional liquid-based Li-ion energy storage systems. Moreover, the employment of SSEs can enhance the packing density of Li-ion batteries, mitigate the issues of self-discharge, and potentially lead to longer life expectancy and improved cycling performance. In this context, Li garnets [1–8], LISICON, thio-LISICON [9–11], Li_2_S–P_2_S_5_ glass [12], Li_7_P_3_S_11_ glass-ceramics [13], and argyrodites Li_6_PS_5_X (X = Cl, Br, I) [14,15] have recently emerged as SSE battery candidates. In particular, garnet-type Li_7_La_3_Zr_2_O_12_ (LLZO) SSEs show great promise and might be the eventual solution for commercialization of solid-state Li-ion battery. LLZO possess an advanced set of properties, such as high ionic conductivity of up to 1 mS cm^−1^ (RT) [1,2,16,17], relatively low level of electronic conductivity of *ca*. 10^−8^ S cm^−1^ (RT) [18,19], wide electrochemical window (>6 V vs Li/Li^+^ obtained in the experiments) [20], and high chemical stability with metallic lithium [21,22].

In this perspective, we discuss the current understanding and the pursuits in the design of solid-state batteries based solely on LLZO electrolytes towards their fabrication with competitive energy and power densities. In the first part of this work, we look into the reported methodologies for making LLZO all-solid-state cathodes and discuss their pros and cons. We then turn to Li plating and stripping processes at the LLZO/Li interface and discuss various anode designs/strategies enabling us to maintain Li/LLZO contact upon battery cycling and mitigate the formation of the Li dendrites. We then examine different fabrication methodologies of LLZO solid-state electrolyte and analyze different contributions to the energy densities of Li-garnet solid-state batteries (SSBs), such as the LLZO thickness, the thickness of the composite cathode (cathode areal capacity), and the content of LLZO in composite cathodes. To this end, we survey the prospects of Li-garnet SSBs, emphasizing the practical hurdles that remain to be overcome.

## Feasibility of making of LLZO-based solid-state cathode

**Figure 1.**
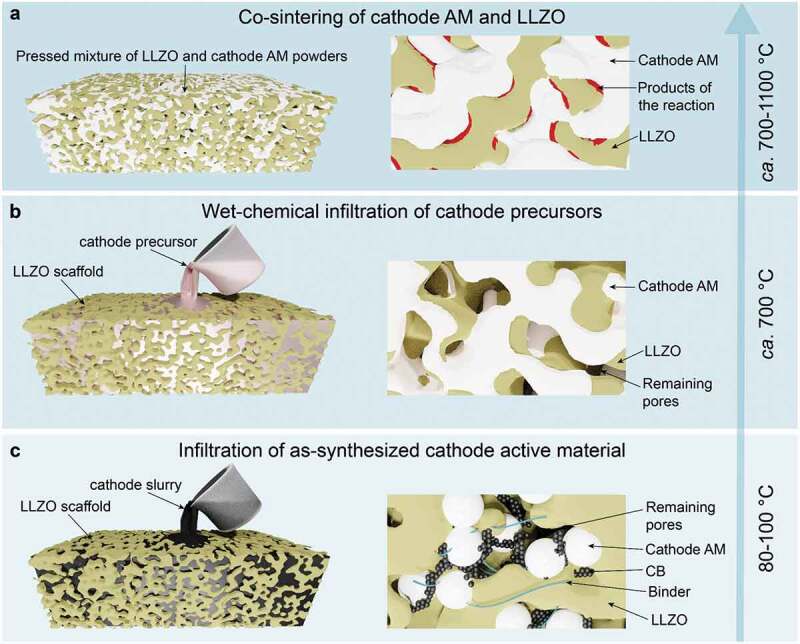
Summary of different fabrication approaches of LLZO-based solid-state cathodes.

Although all-solid-state batteries are often seen as a barely adjusted version of liquid-based Li-ion systems, where liquid electrolyte is replaced by a solid-state counterpart, adoption of solid-state design is exceedingly complex as it requires a vastly different fabrication methodology. With respect to the fabrication of all-solid-state cathodes in combination with LLZO SSE, diverse approaches have been tested so far, which fall into three categories (Figure 1): co-sintering of LLZO with active cathode materials, with or without sintering additives, at elevated temperatures (*ca*. 1100°C), (ii) wet-chemical infiltration of cathode precursors into as-sintered LLZO scaffold, with their subsequent annealing at intermediate temperatures (*ca*. 700°C), and (iii) infiltration of the as-synthesized cathode material in as-sintered LLZO scaffold. Of note, the thin-film approach (usually <1 μm electrodes) has not been considered in this work as it is attractive only for specific applications, such as medical devices and sensors. Still, it is unpractical for mobility due to the limited thickness of the electrode layer yielding low areal cathode capacities. On the contrary, the blended approach allows the fabrication of electrodes with much higher areal charge-storage capacity.

As for the first methodology based on co-sintering, the analysis of the literature highlights potential obstacles to its possible employment. That stems from poor chemical compatibility and maintenance of the phase stability of LLZO electrolytes with current cathode chemistries at high temperatures of 500–700°C or higher, leaving no room for co-sintering to gain sufficient mechanical strength. For instance, insulating products of the decomposition reaction between LLZO and a LiCoO_2_ (LCO) cathode were detected above 700°C [23]. High-voltage spinel cathodes (Li_2_NiMn_3_O_8_, Li_2_FeMn_3_O_8_, and LiCoMnO_4_) react with LLZO even at 500°C [24]. LiFePO_4_ (LFP) cathodes are challenging to sinter with LLZO, as phase decomposition already takes place above 400°C [25]. As previously reported, the co-sintering temperature of composite LLZO cathode can be lowered to ~700°C in the case of the addition of sintering agents such as Li_3_BO_3_ [26]. However, the presence of borate phases (up to a 35 wt%) reduces the charge-transfer kinetics in the composite cathode due to their lower Li-ion conductivity, eventually decreasing its charge-storage capacity.

To overcome the issues of the chemical compatibility of LLZO with cathode materials upon co-sintering at high temperatures, recently, an alternative design was proposed by Rupp et al. [25]. The approach is based on the synthesis of the cathode particles performed directly in an as-sintered porous LLZO scaffold. In the case of the synthesis of LiCoO_2_ cathode using LiNO_3_ and Co(NO_3_)_2_ as precursors, the processing temperature could be decreased down to 700°C. Aiming to decrease further processing temperatures, recently Doeff et al. [27] proposed infiltrating into as-sintered LLZO scaffold as-synthesized LiNi_0.6_Mn_0.2_Co_0.2_O_2_ (NMC622) particles in the form of slurry with carbon black and pVdF binder. Interestedly, to improve the contact between NMC particles and LLZO surface upon cycling, a plastic-crystal electrolyte based on LiTFSI (4 mol %)/LiBOB (1 mol %)/succinonitrile (SCN) was additionally melt-infiltrated into the LLZO scaffold. The added electrolyte solidifies upon cooling to room temperature and serves as an ionically conducting bridge between the NMC622 and the LLZO framework. Another version of the infiltration approach is the direct melting of cathode materials on the surface of LLZO cathodes (for instance, sulfur), making the infiltration process relatively simple and scalable [28]. However, considering the conversion nature of such materials and associated massive volume changes upon lithiation and delithiation, potentially hindering the cycling stability of battery, the applicability of such solid-state cathodes is still to be proved.

## The design of LLZO/Li anode side

**Figure 2.**
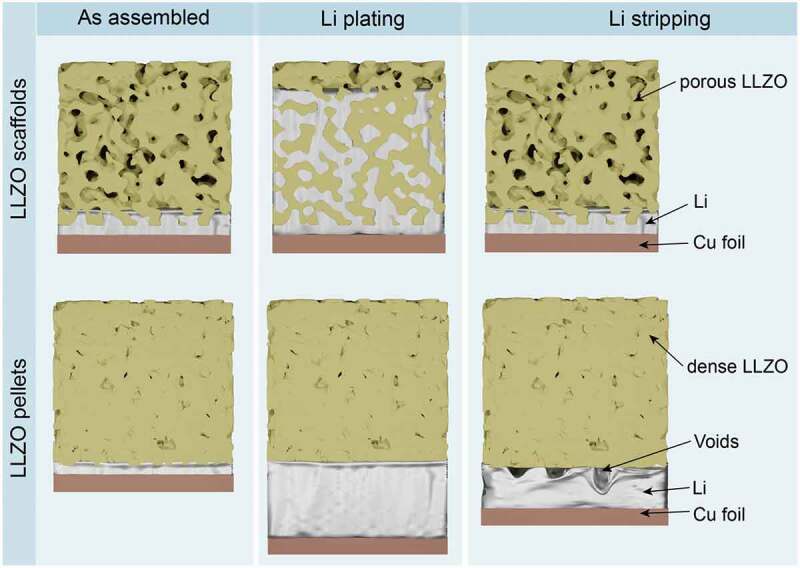
Schematics of Li plating and stripping at dense or porous Li/LLZO interface.

The volume change of the Li metal anode becomes an issue when depositing a high Li areal capacity of 1–5 mAh cm^−2^, which corresponds to a thickness of 5–25 µm. The latter indicates that solid-state battery design should account for the dynamic expansion of the Li metal anode upon charge. Additionally, upon stripping of Li, *i.e*. upon discharge, stack pressure should be applied to the LLZO/Li interface to prevent the formation of voids caused by the insufficient Li^+^ diffusion and utilized pressure for the replenishment of the Li, which is dissolved into LLZO [29]. Otherwise, voids might accumulate at the LLZO/Li interface, leading to an increase in local current density and consequently to the formation of Li dendrites during cycling (Figure 2). As indicated by Bruce et al. [30], to achieve stable cycling at current densities exceeding 1 mA cm^–2^, applied stack pressure should be *ca*. 10 MPa. However, the external stack pressure is a double-edged sword that can also lead to faster cell failure due to increased mechanical stress [31–33]. A possible solution to account for the dynamic changes in lithium thickness is the employment of a scaffold-type LLZO structure since, in this case, no stacking pressure is required. Upon Li plating, Li can be stored in the pores of the LLZO scaffold, resulting in no dynamic change of the cell volume. Furthermore, during stripping, the formation of voids is mitigated by the large surface area of the LLZO/Li interface (Figure 2). Another key feature of the porous LLZO structures is the potential to increase the critical current density of Li plating (up to 10 mA cm^−2^), i.e. the current density at which Li dendrites do not form [28].

Apart from the issues of volume change of Li upon its plating and stripping and associated challenges in the design of the anode part of the solid-state battery, the poor LLZO wettability by lithium metal is another matter that should also be considered [34]. It is mainly caused by the Li-ion-insulating layer at the LLZO surface, composed of LiOH and Li_2_CO_3_ [33,34], although some differences in its constituents have been reported [35–37]. The presence of this layer between LLZO and Li metal has decisive implications on the electrochemical performance of Li-garnet SSBs. Mainly, this causes an increase in the Li/LLZO interfacial resistance [35,36] and, consequently, high-voltage polarization upon Li plating/stripping [38]. Additionally, it might result in the formation of Li dendrites induced by the inhomogeneous distribution of the applied current density [39]. Aiming to solve the issue of poor LLZO wettability by lithium metal, various methods of LLZO surface treatment were comprehensively examined. Namely, conventional heat treatments of LLZO at 600–900°C [40] and surface treatments by LiBF_4_ in ACN [41] or HCl [42] have been demonstrated to be efficient in removing Li-ion insulating surface layers. Moreover, the employment of Sb [43], Mg [44], Ge [45], Al_2_O_3_ [34], Au [16], SnO_2_ [46], and graphite [47] as interlayers between LLZO and Li was shown to enable the reduction of the Li/LLZO interfacial resistance.

## The aspects of LLZO SSE fabrication

**Figure 3.**
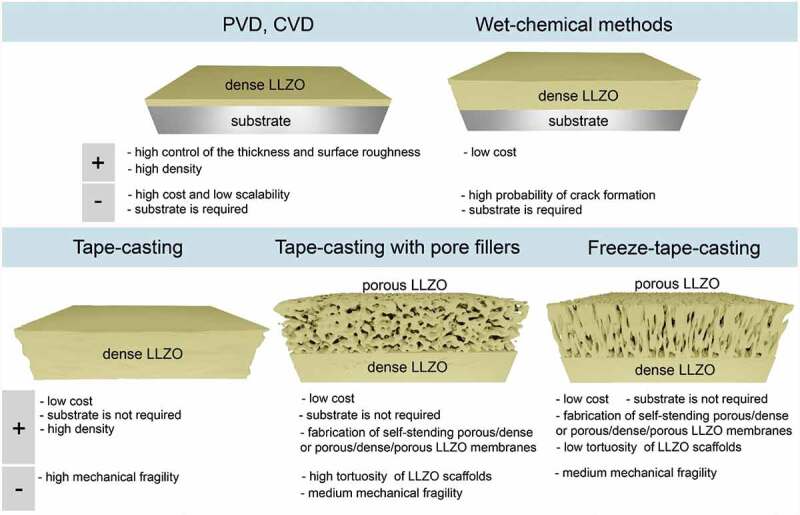
Overview of different methods of LLZO SSE fabrication and the respective advantages and disadvantages of each approach.

In principle, having already self-standing LLZO-based cathode membranes, LLZO solid-electrolyte layer can be made in the form of thin film by physical vapor deposition (PVD) or chemical vapor deposition (CVD) processes (Figure 3). However, it is unlikely that these methods will be used to fabricate commercial solid-state batteries, as the cost and scalability aspects of PVD and CVD are questionable. Given the high investment required to scale up the production of such films, they have mainly been used as model systems to study the reactions at the electrode–electrolyte interface, but will most likely not find a real entry into commercial SSB designs.

Apart from PVD and CVD deposition techniques, LLZO SSE can also be prepared by wet-chemical techniques, enabling obtaining LLZO solid-electrolyte layer with the desired electrolyte thickness, ranging from the submicrometre level to several micrometers (Figure 3). Although, as in the case of PVD and CVD methods, one requires appropriate anode or cathode substrates, wet-chemical synthesized films are attractive for manufacturing cost reduction and scale-up of SSB electrolytes, obviating the need for an expensive vacuum chamber or high-energy source. Conventionally, wet-chemical ceramic processing is based on spray pyrolysis or sol–gel synthesis combined with dip coating, or spin-coating routes. One of the main challenges of these techniques is to gain control of drying of the Li-ceramic film without crack formation during deposition and post-annealing and sintering. Additionally, heat-treatment and sintering on their own could be problematic considering the chemical reaction of the cathode with LLZO, as discussed above.

Considering the necessity of the cathodic or anodic substrates when employing wet-chemical methods, the technology based on direct use of ceramic LLZO powders enabling the fabrication of free-standing membranes is considered as one of the most prominent fabrication strategies for the industry. This approach involves the tape casing of as-synthesized LLZO powders in the form of slurry on the substrate, similar to the process used for the fabrication of graphite and cathode materials in conventional battery manufacturing. After the drying, the substrate can be removed from the substrate by mechanical delamination. This method has been used to achieve a thickness of LLZO membranes as low as 15 μm after sintering [48]. Importantly, tape casting methodology can be used for making monolith porous/dense/porous LLZO membranes (Figure 3), wherein both porous layers can be used for cathode particles or Li metal anode infiltration. The porous layers could be fabricated similarly to dense LLZO part, but employing pore fillers, burned during the sintering process [28]. The porosity of the scaffold part can be controlled by the size of the polymer-based pore formers such as poly(methyl methacrylate) spheres. Alternatively, scaffold structures can be fabricated using a freeze tape casting process (FTC) [27]. In this case, a tape-casted LLZO coating is prepared using conventional ceramic slurry using CO_2_ ice crystals as pore fillers and transferred immediately to a cold surface. The latter causes phase separation and solidification of the solvent, which can be sublimed under vacuum, yielding a vertically aligned porous structure, being ready for sintering. FTC is a scalable method for making thin porous films, with high control over total pore volume, pore size, and morphology. A key feature of the FTC prepared LLZO scaffolds is the low tortuosity (approaching unity) pore channels along the thickness direction, which aids infiltration and shortens Li diffusion path lengths. Furthermore, structurally resilient FTC-LLZO scaffolds can be readily prepared at very high porosities in the green state, exceeding 90%, compared to methods using traditional pore-forming fugitives. The FTC process is also environmentally friendly as ice crystals function as the pore-former, not polymers, which generate CO_2_ during burnout. Comparisons of LLZO membranes prepared by tape casting and freeze tape casting methods, as well as the electrochemical performance of the corresponding membrane-based full cells, can be found in the Supporting information (see Table S1).

Although direct employment of LLZO powders in the form of dense, porous/dense, or porous/dense/porous membranes seems the most feasible method for industrial fabrication of Li-garnet SSBs, the fabrication of commercial size ceramic LLZO membranes (credit card format or larger) is a very challenging task. These difficulties are mainly related to maintaining the flatness of the membranes and the correct Li stoichiometry on their surface during sintering. Unlike pellets, where mechanical polishing can remedy these problems, self-standing LLZO membranes cannot withstand such a post-fabrication process.

## Energy density of Li-garnet solid-state batteries

**Figure 4.**
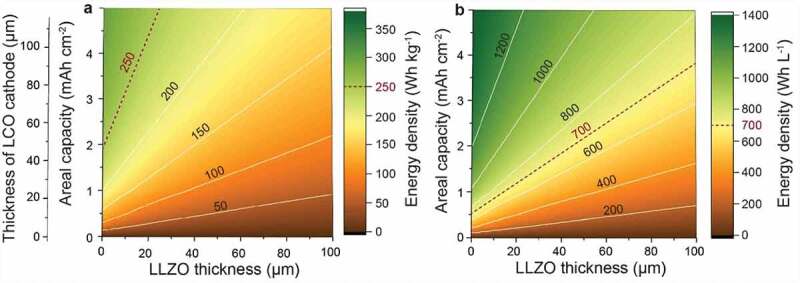
Calculated gravimetric (a) and volumetric (b) energy densities of Li-garnet SSBs *vs*. cathode areal capacity and LLZO SSE thickness. The battery is composed of Li metal anode, LLZO SSE, and LiCoO_2_/LLZO cathode. Reproduced from Ref [52] with the permission of, ACS.

To achieve the maximum energy density of Li-garnet SSBs, the same considerations should be made that apply to conventional Li-ion batteries. These include minimizing the thickness of the electrolyte layer and increasing the area capacities of the cathode and anode. As indicated by Janek [49], Wachsman [28], and others [50,51], the use of LLZO SSE with a thickness of 20–50 µm is mandatory to achieve the gravimetric energy densities of conventional Li-ion batteries. Recently, Kravchyk et al. [52] calculated the LLZO thicknesses of all-solid-state LLZO batteries, which enable the cell to achieve the energy density of a conventional LIB (250 Wh kg^–1^ and 700 Wh L^–1^) for a given cathode composition and cathode areal capacity. For instance, in the case of a LLZO all-solid-state system with a conventional LCO cathode (97 wt% LCO, 3 wt% CB, 1 wt% pVdF, 30% porosity), where hypothetically all pores have been replaced by LLZO SSE, low LLZO thicknesses of 13–25 μm must be used in combination with 3.5–5 mAh cm^−2^ electrodes to achieve the energy density of 250 Wh kg^−1^ (Figure 4). Lower cathode loadings of <3.5 mAh cm^−2^ would require even thinner LLZO membranes. For LiNi_0.33_Mn_0.33_Co_0.33_O_2_ (NMC111) and LFP cathodes, LLZO thickness range is similar, 18–32 µm and 4–12 µm. Only high-voltage LiNi_0.5_Mn_1.5_O_4_ (LMNO) cathode or conversion-type materials such as FeF_3_ and S might allow using higher thicknesses of 38–60 µm, 34–55 µm, and 35–56 µm at identical volumetric LLZO content in the cathode (30 vol%), accordingly. Importantly, considering the previously stated DOE performance goal, targeting the energy density of 350 Wh kg^−1^, the LLZO electrolytes should be even thinner, at the level of 9 µm, 28 µm, and 29 µm for LMNO, FeF_3_, and S cathodes, respectively, considering the cathode areal capacity of 5 mAh cm^−2^.

Another factor being critically important in the fabrication of LLZO-based cathodes is finding a minimal yet sufficient LLZO content in the composite cathodes. Thus far, the volumetric content of pores in conventional cathode was optimized at the level of 20–30 vol%, being sufficient to provide efficient transport of Li^+^ ions, when filled with a liquid electrolyte. This content might not be enough in the case of the employment of LLZO SSE considering lower Li-ion conductivity of LLZO. An overview of the maximal content of LLZO for LCO, NMC111, and LFP composite cathodes, which still permits matching the energy densities of the state-of-the-art Li-ion batteries (250 Wh kg^–1^), is given in Ref [52]. For instance, supposing that the Li-garnet SSB has a LLZO thickness as low as 10 μm and an areal capacity of 5 mAh cm^–2^, solid composite cathodes cannot have more than 40–46 wt% LLZO to result in gravimetric energy densities that at least match those of conventional Li-ion batteries. With regard to the volumetric energy density of 700 Wh kg^–1^, the maximum permitted LLZO volume content lies in the range of 45–68 vol%. Notably, in the case of higher thickness of LLZO and lower cathode areal capacities, the lower content of LLZO in the composite electrode should be used.

## Conclusions and outline

The field of Li-garnet SSBs has made great strides; however, this technology still requires continued advances on multiple fronts, including design, mechanical brittleness, and safety.

**Figure 5.**
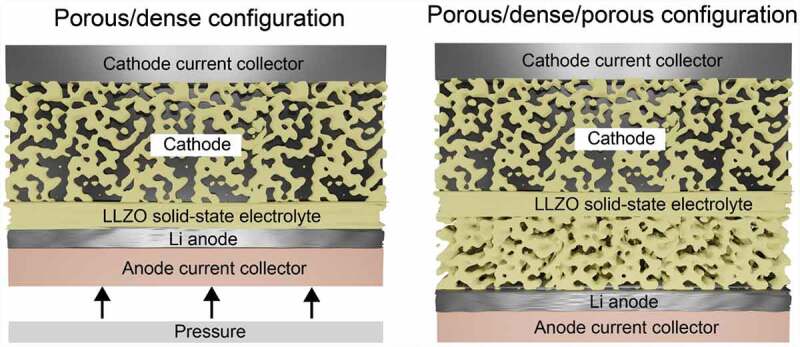
Comparison of two configurations of Li-garnet SSB based on porous/dense and porous/dense/porous LLZO self-standing membranes.

The final design of LLZO solid-state battery should still be determined. Considering the issues of the volume changes of Li upon plating/stripping as well as the compatibility issues of present cathode chemistries with LLZO at high temperature, it seems that the fabrication of the battery based on self-standing three-layer porous/dense/porous LLZO membranes is one of the most feasible design approaches (Figure 5). The use of bilayer porous/dense LLZO membranes with a Li metal anode facing the dense LLZO side is less realistic, as this requires the employment of relatively high pressure of *ca*. 10 MPa. However, a careful analysis of the published data on three-layer structures indicates that several issues remain to be resolved. In particular, the reported thicknesses of the porous and dense LLZO layers are still relatively thick, which significantly reduces achievable the gravimetric and volumetric energy density of Li-garnet SSBs at the cell level. Another issue is related to the difficulty of getting 80–100% of pore occupancy of LLZO scaffold by cathode particles, and it is not yet clear whether it is possible at all. In this context, the employment of cathode with low melting temperature such as S seems a feasible approach. On the other hand, the issue of the low cycling stability of LLZO/sulfur cathode, associated with the volume changes of S, might be challenging to resolve.

One of the pending concerns of Li-garnet SSBs is the brittleness of the LLZO and low fracture toughness, which may require specific technological solutions in handling thin LLZO membranes of large size and placing them in layers. Thus far, the research is carried out using one layer of 1-cm^2^-sized membranes at maximum. In this context, unlike current liquid electrolyte systems, the manufacturability and material component costs of such a solution are unknown, and thus the value of these features will need to be weighed accordingly with any added cost.

Although Li-garnet SSBs are intrinsically safe in the discharge state, certain safety issues are possible when the batteries are charged. In this context, catastrophic failure modes such as shorting should be investigated using Li-garnet SSBs of commercial size using testing protocols comparable to standard liquid-based Li-ion systems.

## Supplementary Material

Supplemental Material
