# Supplementary material for: Perspective on design and technical challenges of Li-garnet solid-state batteries
Source: Sci Technol Adv Mater. 2022 Jan 18;23(1):2018919. doi: 10.1080/14686996.2021.2018919 (PMC8774065; doi:10.1080/14686996.2021.2018919)
Supplement: Supplemental Material [file TSTA_A_2018919_SM9732.docx]

*Supporting Information for*

Perspective on design and technical challenges of Li-garnet solid-state batteries

*Kostiantyn V. Kravchyk^a,b*^ and Maksym V. Kovalenko^a,b*^*

^a^Laboratory for Thin Films and Photovoltaics, Empa – Swiss Federal Laboratories for Materials Science and Technology, Überlandstrasse 129, CH-8600 Dübendorf, Switzerland; ^b^Laboratory of Inorganic Chemistry, Department of Chemistry and Applied Biosciences, ETH Zürich, Vladimir-Prelog-Weg 1, CH-8093 Zürich, Switzerland

Corresponding Authors:

*E-mails: [mvkovalenko@ethz.ch](mailto:mvkovalenko@ethz.ch) and [kravchyk@inorg.chem.ethz.ch](mailto:kravchyk@inorg.chem.ethz.ch)

**Table S1.** The comparison of LLZO membranes prepared by tape casting and freeze tape casting methods, as well as the electrochemical performance of corresponding membrane-based full cells.

| Reference | Type of LLZO membrane | Thickness, µm | Fabrication method | Cathode material | Cathode active material loading, mg cm^-2^ | Current density (mAh g^-1^ or mAh cm^-2^) | Initial capacity (mAh g^-1^) | Retained capacity (mAhg^-1^) | Cycle number | Potential range (V vs. Li^+^/Li) |
| --- | --- | --- | --- | --- | --- | --- | --- | --- | --- | --- |
| [1] | dense/porous | 70 µm (porous)  35 µm (dense) | Tape casting | S | 7.5 mg cm^-2^ | 0.2 mA cm^-2^ | 645 mAh g^-1^ | 400 mAh g^-1^ | 32 | 1.0-3.5 V |
| [2] | porous/dense/porous | 49 µm (porous)  28 µm (dense)  47 µm (porous) | Tape casting | - | - | - | - | - | - | - |
| [3] | dense/porous | 50 µm (porous)  20 µm (dense) | Tape casting | NMC622 | 14 mg cm^-2^ | 0.2 mA cm^-2^ | 150 mAh g^-1^ | 175 mAh g^-1^ | 28 | 2.5-4.5 V |
| [4] | porous/dense/porous | 50 µm (porous)  15 µm (dense)  50 µm (porous) | Tape casting | S | 5.4 mg cm^-2^ | 50 mA g^-1^ | 1200 mAh g^-1^ | 1100 mAh g^-1^ | 50 | 1.5-3.5 V |
| [5] | dense/porous | 60 µm (porous)  30 µm (dense) | Tape casting | S | - | 1 mA cm^-2^ | 3 mAh cm^-2^ | 3 mAh cm^-2^ | 50 | 2.1-2.45 V |
| [6] | dense/porous | 200 µm (porous)  20 µm (dense) | Freeze tape casting | NMC622 | 15.7 mg cm^-2^ | 0.3 mA cm^-2^ | 175 mAh g^-1^ | 125 mAh g^-1^ | 90 | 2.5-4.7 V |
| [7] | porous/dense/porous | 70 µm (porous)  20 µm (dense)  70 µm (porous) | Tape casting | S | 5.3 mg cm^-2^ | 0.22 mA cm^-2^ | 1244 mAh g^-1^ | 1200 | 8 | 1-3 V |
| [8] | porous | 50 µm (porous) | Freeze tape casting | - | - | - | - | - | - | - |
| [9] | porous/dense/porous | 130 µm (porous)  37 µm (dense)  130 µm (porous) | Freeze tape casting | NMC622 | 4-5 mg cm^-2^ | 17.5 mA g^-1^ | 135 mAh g^-1^ | 125 mAh g^-1^ | 4 | 2.5-4.4 V |
| [10] | porous/dense/porous | 60 µm (porous)  30 µm (dense)  60 µm (porous) | Tape casting | S | - | 83.75 mA g^-1^ (60°C) | 500 mAh g^-1^ | 800 mAh g^-1^ | 5 | 1.4-3 V |

**References**

1. Fu K, Gong Y, Hitz GT, et al. Three-dimensional bilayer garnet solid electrolyte based high energy density lithium metal–sulfur batteries. Energy Environ Sci. 2017;10(7):1568-1575.

2. Yang C, Zhang L, Liu B, et al. Continuous plating/stripping behavior of solid-state lithium metal anode in a 3D ion-conductive framework. Proc Natl Acad Sci. 2018;115(15):3770-3775.

3. Liu B, Zhang L, Xu S, et al. 3D lithium metal anodes hosted in asymmetric garnet frameworks toward high energy density batteries. Energy Storage Mater. 2018 2018/09/01/;14:376-382.

4. Xu S, McOwen DW, Zhang L, et al. All-in-one lithium-sulfur battery enabled by a porous-dense-porous garnet architecture. Energy Storage Mater. 2018 2018/11/01/;15:458-464.

5. Xu S, McOwen DW, Wang C, et al. Three-dimensional, solid-state mixed electron–ion conductive framework for lithium metal anode. Nano Lett. 2018 2018/06/13;18(6):3926-3933.

6. Shen H, Yi E, Amores M, et al. Oriented porous LLZO 3D structures obtained by freeze casting for battery applications. J Mater Chem A. 2019;7(36):20861-20870.

7. Hitz GT, McOwen DW, Zhang L, et al. High-rate lithium cycling in a scalable trilayer Li-garnet-electrolyte architecture. Mater Today. 2019 2019/01/01/;22:50-57.

8. Shen H, Yi E, Heywood S, et al. Scalable freeze-tape-casting fabrication and pore structure analysis of 3D LLZO solid-state electrolytes. ACS Appl Mater Interfaces. 2020 2020/01/22;12(3):3494-3501.

9. Yi E, Shen H, Heywood S, et al. All-solid-state batteries using rationally designed garnet electrolyte frameworks. ACS Appl Energy Mater. 2020 2020/01/27;3(1):170-175.

10. Xie H, Yang C, Ren Y, et al. Amorphous-carbon-coated 3D solid electrolyte for an electro-chemomechanically stable lithium metal anode in solid-state batteries. Nano Lett. 2021 2021/07/28;21(14):6163-6170.
